# Are tomorrow's dentists ready for a sustainable future? Insights from a European student survey

**DOI:** 10.3389/fdmed.2026.1788789

**Published:** 2026-06-08

**Authors:** Ana Maria Cristina Țâncu, Henk Simon Brand, Marina Imre, Mihaela Pantea, Silviu Mirel Pițuru, Ruxandra Sfeatcu, Laura Iosif, Miruna Dinescu, Bogdan Alexandru Dimitriu, Tudor Claudiu Spînu, Andreea Cristiana Didilescu

**Affiliations:** 1 Department of Prosthodontics, Faculty of Dentistry, “Carol Davila” University of Medicine and Pharmacy, Bucharest, Romania; 2 Department of Oral Biochemistry, Academic Centre for Dentistry Amsterdam (ACTA), Amsterdam, Netherlands; 3 Department of Organization, Professional Legislation and Management of the Dental Office, Faculty of Dentistry, “Carol Davila” University of Medicine and Pharmacy, Bucharest, Romania; 4 Discipline of Oral Health and Community Dentistry, Faculty of Dentistry, “Carol Davila” University of Medicine and Pharmacy, Bucharest, Romania; 5 Department of Endodontics, Faculty of Dentistry, “Carol Davila” University of Medicine and Pharmacy, Bucharest, Romania; 6 Department of Embryology and Microbiology, Faculty of Dentistry, “Carol Davila” University of Medicine and Pharmacy, Bucharest, Romania

**Keywords:** dental education, europeanperspective, future dentists, green dentistry, sustainability

## Abstract

**Introduction:**

The significant environmental impact related to modern dental practices highlights the need for increased awareness and attitude shifts among future European dentists. Therefore, this study aimed to assess dental students’ perceptions and knowledge levels regarding sustainability in oral healthcare across Europe.

**Methods:**

The study was conducted among 175 students from 14 dental schools in 11 European countries, who completed an online questionnaire.

**Results:**

Most respondents were female (71%) vs. male (27%), with a mean age of 21.1 ± 2.9 years (18–33). 56% of the respondents correctly identified all three dental sustainability definitions, with no gender differences. Dental activities perceived as having the highest environmental impact were patient care (single-use cups and materials) (51%), dental equipment (18%), and dental materials (14%), with no significant gender differences. Students’ top strategies to reduce environmental impact of dentistry were reducing plastic disposables (77%), using technologies like 3D printing/CAD-CAM (56%), and digital data management (55%). Dental procedures considered to have the highest carbon footprint were amalgam/composite fillings (44%), radiographs (35%), and acrylic dentures (32%). Additionally, 42% of the students expressed interest in an elective course on sustainability in dental practice.

**Conclusions:**

By exploring future practitioners’ understanding of sustainability, this research could offer perspectives that may be useful in shaping curriculum considerations, fostering environmentally responsible clinical training, and supporting policy dialogue at institutional and European levels.

## Introduction

Sustainability has become a key principle in global policy and education, especially as climate change accelerates. Defined by the Brundtland Commission in 1987 as “development that meets the needs of the present without compromising the ability of future generations to meet their own needs,” sustainability emphasizes long-term environmental stewardship along with social and economic progress (1). This definition highlights essential ideas: the intergenerational equity aspect (future generations), the fulfillment of current needs (the “needs” of the present), and the limits set by the capacities of natural, social, and economic systems to support development (2).

Moreover, sustainability has emerged as a central priority in contemporary healthcare discourse, reflecting global concerns about environmental stewardship, equitable access to care, and long-term health outcomes. The United Nations Sustainable Development Goals (SDGs), as outlined in Transforming our World: the 2030 Agenda for Sustainable Development (3), offer a comprehensive international framework that links health, education, and environmental responsibility. Several SDGs—particularly those addressing good health and well-being (SDG 3), quality education (SDG 4), sustainable consumption and production (SDG 12), and climate action (SDG 13)—are directly relevant to dental practice and professional training, emphasizing the importance of preparing future dentists to contribute to sustainable healthcare systems. In the context of dental education, aligning curricula with these global goals may enhance students' capacity to recognize and respond to the environmental and societal challenges facing oral healthcare.

Dental activities contribute significantly to environmental pollution: waste materials (including single-use personal protective equipment and clinic consumables), potentially toxic materials (e.g., amalgam, chemical disinfectants), high energy and water consumption, and use of paper and administrative resources. The largest contributors to the environmental impact of oral healthcare are patient and staff travel to and from the dental practice, which add to carbon emissions and transportation-related environmental burdens, along with procurement and energy use (4, 5). This means that providing oral healthcare has a considerable environmental footprint (5, 6). Therefore, there is a clear need to identify current drivers, opportunities, recommendations, and best practices that can create change through increased awareness (7–9).

For each of these activities, which carry important environmental impact, there are tangible solutions to reduce the negative impact. One of the starting points should be to establish the degree of awareness on this concept among dental practitioners and the future workforce—namely, current students in dental medicine—because this need is of utmost importance in all sectors of activity (10).

It follows that dental education must address not only clinical competencies and professional ethics, but also, sustainable practice: how to select materials, minimize waste, reduce energy consumption, adopt low-carbon transport and integrate sustainable thinking into daily dental care. A lack of student awareness and knowledge will represent a barrier to achieving these goals; therefore, assessing the current knowledge of dental students is a critical first step.

Sustainability awareness among university students has become a critical area of investigation, as higher education institutions are increasingly expected to integrate environmental, social, and economic considerations into their curricula and campus operations. Studies in several countries among students from different study programs demonstrated varying levels of sustainability literacy, indicating that while many recognize the importance of sustainable development, knowledge of specific practices and strategies remains limited (11–14). These findings highlight the need for targeted educational interventions that not only raise awareness but also equip students with practical skills to implement sustainability principles in both personal and professional contexts. Universities worldwide are increasingly providing resources, guidelines, and curricular modules to facilitate this integration, emphasizing the broader role of higher education in fostering sustainability literacy and responsible citizenship (9–11).

Within the field of dentistry, the imperative for sustainable practice is particularly acute due to the profession's significant environmental footprint. Dental practices contribute to pollution through the disposal of consumables, personal protective equipment, and the use of energy-intensive equipment, as well as through patient and staff commuting (15–19). Recent research underscores that sustainable strategies in dental care, such as reducing waste, optimizing energy use, and adopting resource-efficient practices, can meaningfully decrease the environmental impact of dental services (20–22). Initiatives such as green dentistry advocate for integrating these principles into everyday clinical operations, highlighting the dual benefits of environmental stewardship and professional responsibility (21, 22). Consequently, assessing the level of sustainability knowledge among current dental students is essential, as it represents the foundation for cultivating a workforce capable of implementing environmentally conscious practices throughout their careers (18, 19).

Despite the clear rationale for embedding sustainability knowledge among dental students, recent literature highlights persistent gaps. The 2025 scoping review by Bamedhaf et al. of environmental sustainability in dental curricula found that while awareness of sustainability among students and faculty is growing, the integration of sustainability into curricula remains inconsistent (23). The review reported that many dental curricula lacked structured sustainability content, faculty training and assessment frameworks. In this review, an important finding was that institutional variables, such as dedicated teaching time, resources and faculty engagement, were strongly associated with student exposure to sustainability content.

At the European level, Field et al. presented recommendations for embedding sustainability within oral health professional curricula and noted that the breadth of curriculum integration across European institutions varied substantially (24). Similarly, the ADEE Special-Interest Group report by Dixon et al. identified key challenges: time constraints, faculty expertise gaps, resistance to change and limited learning resources—all of which affect the student learning experience (25). These findings suggest that student knowledge is likely shaped not only by individual interest and prior awareness, but also by the institutional context and curricular architecture.

To date, most studies examining initiatives aimed at increasing sustainability awareness among students and academic staff have been conducted primarily within undergraduate and postgraduate business and economics programmes (26–28). These initiatives commonly emphasize the integration of sustainability into institutional strategic planning, operational practices, campus activities, and student engagement.

Within this context, the present research aims to explore the level of sustainability knowledge and awareness among students of dental schools in Europe. Specifically, this study seeks to examine dental students' understanding of sustainability concepts and could contribute evidence that may inform curriculum development and support institutional efforts to further integrate sustainability into dental education.

## Materials and methods

For the present study, an online questionnaire assessing the attitudes of dental students in Europe toward sustainability was developed and partially derived from a previously published instrument (29), with slight adaptations for use in the present study. The original instrument (29), written in the English language, was developed in consultation with sustainability experts from Bemari.co.uk (a consultancy specializing in sustainability and procurement) and informed the structure and content of several items in the present questionnaire. Although a formal pilot study was not conducted prior to large-scale distribution, the present investigation may be considered an initial exploratory application of this adapted instrument in a European dental student population. The original questionnaire (29) was carefully reviewed by all authors, slightly adjusted to align with the aims of the current study, and minor potential linguistic ambiguities were identified and corrected. The resulting version was subsequently evaluated by a group of ten doctoral dental students, fluent in English, who assessed the clarity and comprehensibility of the items. Based on their feedback, no major modifications were deemed necessary, and the finalized version was used in the present study. The final questionnaire was approved by the Scientific Research Ethics Committee of “Carol Davila” University of Medicine and Pharmacy in Bucharest, Romania (protocol number 33066). The full questionnaire used in this research is presented in the Supplementary Material section of this manuscript.

The first part of the questionnaire collected general information about dental school, gender and study year. In the second part, the perspective of the student on sustainability in dentistry was investigated with 8 multiple choice, rating scale and open questions. The third part contained 4 multiple choice and open questions about the personal sustainability lifestyle of the respondents.

The questions were entered in the internet survey program Qualtrics (Qualtrics, Provo, UT, USA). A link to the questionnaire was distributed in a similar way as previously described (30). In their newsletter, the Association for Dental Education in Europe (ADEE) called on the Deans of the affiliated dental schools to distribute the link among their students. In addition, the European Dental Students Association (EDSA) was approached to distributed the link among students of affiliated dental schools. The questionnaire could be completed between March 10th 2025 and June 16th 2025. Participation was on a voluntary base, and all responses were anonymous. Questionnaires were included for analysis when a respondent completed at least some of the questions about the perspective of the student on sustainability in dentistry. This selection resulted in 175 useful questionnaires.

Data are expressed as percentages or mean ± S.D. The rating scale items were statistically analyzed using IBM SPSS Statistics version 28.0 (IBM Corp, Armonk NY, USA), using Mann–Whitney tests and Chi Square tests. All levels of significance were set at *P* < 0.05.

## Results

The questionnaire was completed by 175 dental students from 14 dental schools in 11 European countries: Cyprus (75 respondents), Denmark (6), France (2 dentals school with 3 respectively 1 respondents), Hungary (1). Italy (1), Lithuania (6), Malta (1), the Netherlands (18), Romania (2 dental school with 45 respectively 1 respondents), Turkey (9) and United Kingdom (2 dental schools with 5 respectively 3 respondents), Most respondents were female (*n* = 125, 71%), followed by male (*n* = 48, 27%), with 2% preferring not to disclose their gender. Approximately three quarter of the students were in the Bachelor phase of their study (*n* = 129, 74%). The mean age of the respondents was 21.1 ± 2.9 years (range 18–33 years).

More than half of the respondents correctly identified all three dental sustainability definitions presented in the questionnaire, and no statistically significant differences were observed with respect to gender or stage of study (Table 1).

**Table 1:** European dental students’ selected responses regarding definitions of sustainability relevant to the dental profession.

Dental students' s answers	Total (*n* = 175)	Women (*n* = 129)	Men (*n* = 46)	Bachelor (*n* = 129)	Master (*n* = 46)
Meeting the needs of the dental patients and population of the present without compromising the ability of future generations to meet their own needs	59 (34%)	41 (33%)	16 (33%)	43 (33%)	16 (35%)
Taking care of the three “pillars”, the environment, society, and the economy, reliant on each other and interconnected	17 (10%)	13 (10%)	4 (8%)	9 (7%)	8 (17%)
Minimizing the health-related impact of climate change and environmental degradation	4 (2%)	3 (2%)	1 (2%)	2 (2%)	2 (4%)
All above	95 (54%)	68 (54%)	27 (56%)	75 (58%)	20 (44%)

Chi-square Women vs. men *p* = 0.979, Bachelor vs. Master *p* = 0.097.

The respondents considered it important for dentists to be aware of sustainability in their sector (mean 7.8 ± 1.9 (range 1–10). No statistically significant difference was observed between male and female students (7.4 ± 2.2 vs. 8.0 ± 1.8, *p* = 0.148), but bachelor students considered this more important than master students (8.0 ± 1.7 vs. 7.2 ± 2.2, *p* = 0.029).

Patient care (e.g., single-use cups and materials) was by far considered the dental activity with the highest environmental impact, followed by dental equipment and dental materials (Table 2). Gender and study phase had no significant effect on this opinion.

**Table 2:** Dental activity with the highest adverse environmental impact, according to European dental students’ responses.

Dental students' s answers	Total (*n* = 158)	Women (*n* = 115)	Men (*n* = 43)	Bachelor (*n* = 115)	Master (*n* = 43)
Use of specialist materials	22 (14%)	17 (15%)	5 (12%)	17 (15%)	5 (12%)
Dental equipment	29 (18%)	25 (21%)	4 (9%)	25 (22%)	4 (9%)
Patients care aspects such as single-use cups and materials	81 (51%)	55 (48%)	26 (60%)	55 (48%)	26 (61%)
Patient paperwork & records	13 (8%)	9 (8%)	4 (9%)	9 (8%)	4 (9%)
Dental office lighting & energy	6 (4%)	5 (4%)	1 (2%)	5 (4%)	1 (2%)
Commute and use of transport to and from the dental office by the patients	5 (3%)	3 (3%)	2 (5%)	3 (3%)	2 (5%)
Other	2 (1%)	1 (1%)	1 (2%)	1 (1%)	1 (2%)

Chi-square. Women vs. Men: *p* = 0.536, Bachelor vs. Master: *p* = 0.519.

Students' top strategies to reduce environmental impact of dentistry were reducing plastic disposables, using technologies like 3D printing/CAD-CAM and digital data management (Table 3). Gender influenced the opinion about the possible effect of the use of dental materials, whereby male student more often assume that these are effective in improving environmental impact. Bachelor students have more confidence that use of modern techniques and technologies may be effective in improving environmental impact than master students. There was also a trend towards statistical significance for the effect of reducing the use of plastic medical disposals, where master students believe that this improves environmental impact.

**Table 3:** Activities considered the most effective in improving environmental impact, according to European dental students’ responses.

Dental students' s answers	Total (*n* = 152)	Women (=107)	Men (*n* = 45)	Chi-square *p*-value	Bachelor (=111)	Master (*n* = 41)	Chi-square *p*-value
Use of modern dental materials	45 (30%)^a^	26 (24%)	19 (44%)	0.016	33 (30%)	12 (29%)	0.956
Use of modern techniques and technologies (3D printing, CAD/CAM, etc.)	85 (56%)	60 (56%)	25 (58%)	0.817	68 (61%)	17 (42%)	0.029
Digital data management & records	84 (55%)	62 (58%)	21 (49%)	0.310	58 (52%)	26 (63%)	0.219
Reducing the use of plastic medical disposals	117 (77%)	85 (79%)	30 (70%)	0.205	81 (73%)	36 (88%)	0.054
Reducing water and electricity consumption	49 (32%)	34 (32%)	13 (30%)	0.854	37 (33%)	12 (29%)	0.634
Educating staff and patients	79 (52%)	57 (53%)	21 (49%)	0.623	58 (52%)	21 (51%)	0.910
Other	3 (2%)	3 (3%)	0 (0%)	0.267	1 (1%)	2 (5%)	0.118

a Total percentages in each column may exceed 100%, as students were invited to select 3 options.

Dental procedures considered to have the highest carbon footprint were amalgam/composite fillings, radiographs, and acrylic dentures (Table 4). Bachelor students reported much more often than master students that they did not know which dental procedures have the largest footprint than master students, while more master students consider porcelain crown the dental procedure with the largest carbon footprint. Trends toward statistically significant differences were observed for amalgam and composite fillings, radiographs, fluoride varnish and precious metal crowns. More master students considered amalgam and composite fillings to have the largest carbon footprint, while more bachelor student considered fluoride varnish and precious metal crown to have the largest carbon footprint. Compared to male students, more female students considered radiographs to have the largest carbon footprint.

**Table 4:** Dental procedures with the largest carbon footprint, according to European dental students.

Dental students' s answers	Total (*n* = 142)	Women (*n* = 99)	Men (*n* = 41)	Chi Square *p*-value	Bachelor (*n* = 103)	Master (*n* = 39)	Chi square *p*-value
Scale and polish	19 (13%)^a^	12 (12%)	7 (17%)	0.436	14 (14%)	5 (13%)	0.904
Amalgam and composite fillings	63 (44%)	45 (46%)	17 (42%)	0.665	41 (40%)	22 (56%)	0.075
Acrylic dentures	45 (32%)	31 (31%)	12 (29%)	0.811	34 (33%)	11 (28%)	0.583
Radiographs	49 (35%)	39 (39%)	10 (24%)	0.090	36 (35%)	13 (33%)	0.856
Extractions	4 (3%)	3 (3%)	1 (25)	0.848	4 (4%)	0 (0%)	0.212
Non-precious metal crowns	21 (15%)	16 (16%)	4 (10%)	0.324	13 (13%)	8 (21%)	0.237
Fluoride varnish	18 (13%)	12 (12%)	6 (15%)	0.686	16 (16%)	2 (5%)	0.096
Endodontic treatment	38 (27%)	29 (29%)	9 (22%)	0.374	24 (23%)	14 (36%)	0.130
Precious metal crowns	38 (27%)	23 (23%)	15 (37%)	0.106	32 (31%)	6 (15%)	0.060
Metal dentures	52 (37%)	33 (33%)	19 (46%)	0.147	34 (33%)	18 (46%)	0.147
Fissure sealants	5 (4%)	4 (4%)	1 (2%)	0.642	4 (4%)	1 (3%)	0.703
Porcelain crowns	30 (21%)	19 (19%)	10 (24%)	0.490	17 (17%)	13 (33%)	0.028
I don’t know	44 (31%)	31 (31%)	12 (29%)	0.811	40 (39%)	4(10%)	0.001

a Total percentages in each column may exceed 100%, as students were invited to select 3 options.

Almost half of the students (42%) expressed interest in an elective course on sustainability in dental practice. No significant differences were observed between men and women, or between bachelor and master students.

Regarding respondents' lifestyle, the majority reported being concerned about environmental issues and having implemented certain behavioral changes; however, they indicated that they would be willing to adopt further measures if provided with additional guidance (Table 5). The percentage of women who chose this option was significantly higher than that of men (*p* *=* *0.041*). Men as well as master students significantly more often chose the option “I am very active in supporting social causes that are important to me” than women and bachelor students, respectively (*p* *=* *0.043*).

**Table 5:** Environmental concern and lifestyle behaviors among respondents.

Dental students' s answers	Total (*n* = 124)	Women (*n* = 85)	Men (*n* = 37)	Chi square *p*-value	Bachelor (*n* = 91)	Master (*n* = 33)	Chi square *p*-value
I am aware of my environmental impact, and it is a big part of all my decisions	25 (20%)^a^	16 (19%)	9 (24%)	0.489	21 (23%)	4 (12%)	0.179
I am very active in supporting social causes that are important to me	23 (19%)	12 (14%)	11 (30%)	0.043	13 (14%)	10 (30%)	0.043
I am worried about the environment, and I have made some changes in my lifestyle, however, I probably could to more if I knew what else to do	86 (69%)	64 (75%)	21 (57%)	0.041	64 (70%)	22 (67%)	0.696
I am not doing anything about my environmental impact, but I would like some help to get started	16 (13%)	11 (13%)	4 (11%)	0.742	13 (14%)	3 (9%)	0.446
I am not concerned about my environmental impact	6 (5%)	3 (4%)	3 (8%)	0.282	5 (6%)	1 (3%)	0.572

a Total percentage in each column may exceed 100%, as students were allowed to select more than 1 option.

The most frequent environmental choices made by dental students in their private lives are turning off lights when not using them, walk or cycle to work or university and recycle waste (Table 6). Gender had a statistically significant effect on the use of energy-efficient light bulbs, which are more often used by male students (*p* *=* *0.020*). On the other hand, waste was more often recycled by female students. A trend toward statistical significance was observed for more women buying organic food. Master students more often used public transport and energy-efficient light bulbs than bachelor students. A trend towards statistical significance for more often recycling waste by master students was also observed.

**Table 6:** Environment-related choices regularly made by participating European dental students.

Dental students' s answers	Total (*n* = 121)	Women (*n* = 82)	Men (*n* = 37)	Chi square *p*-value	Bachelor (*n* = 89)	Master (*n* = 32)	Chi square *p*-value
Walk or cycle to work/university campus	82 (68%)^a^	58 (71%)	23 (62%)	0.353	63 (71%)	19 (59%)	0.236
Use only public transport	41 (34%)	29 (35%)	11 (30%)	0.547	23 (26%)	18 (56%)	0.002
Turn off the lights when I’m not using them	100 (83%)	70 (85%)	29 (78%)	0.345	74 (83%)	26 (81%)	0.808
Use energy-efficient light bulbs	49 (40%)	28 (34%)	21 (57%)	0.020	31 (35%)	18 (56%)	0.034
Buy organic food	29 (24%)	23 (28%)	5 (14%)	0.084	20 (23%)	9 (28%)	0.521
Recycle waste	72 (60%)	54 (66%)	16 (43%)	0.020	49 (55%)	23 (72%)	0.096
Buy less	64 (53%)	44 (54%)	19 (51%)	0.815	47 (53%)	17 (53%)	0.975
Buy used/refurbished goods	37 (31%)	26 (32%)	11 (30%)	0.829	26 (29%)	11 (34%)	0.587
Other	12 (10%)	7 (9%)	5 (14%)	0.404	8 (9%)	4 (13%)	0.569

a Total percentage in each column may exceed 100%, as students were allowed to select more than 1 option.

## Discussions

The current multicenter survey provides contemporary insights into European dental students' awareness, perceptions, and behaviors regarding sustainability in dentistry. Overall, the results indicate that students recognize the relevance of sustainability to clinical practice, yet reveal considerable variability in knowledge, perceived environmental impacts, and confidence in implementing eco- friendly strategies. These findings are broadly consistent with recent literature highlighting that sustainability is increasingly recognized in healthcare education but remains insufficiently integrated into dental curricula across Europe (29–32).

### Understanding of sustainability concepts

More than half of respondents (55%) correctly recognized all sustainability definitions presented in the questionnaire, suggesting gradual improvement in conceptual literacy compared with previous reports where dental students demonstrated limited familiarity with sustainability frameworks (33, 34). Overall, no significant differences were observed based on gender or study phase, which may reflect the notion that sustainability awareness is shaped more by educational exposure than by demographic variables. However, previous research has identified a growing trend of higher female participation in volunteer and social engagement activities, suggesting a potential gender-related tendency toward greater involvement in sustainability-related efforts (35, 36). Nonetheless, the finding that Bachelor students rated the importance of sustainability significantly higher than Master students may reflect generational shifts or heightened curricular exposure during preclinical years. Conversely, a recent study (37) identified age as the only statistically significant demographic predictor of knowledge and practice related to green dentistry among undergraduate dental students; higher knowledge scores were associated to greater awareness, which in turn was significantly influenced by both age and prior training favoring older participants. Another study (38) reported that participating students exhibited only limited to minimal familiarity with the concept of sustainable education, whereas educators demonstrated a clearer understanding of the topic. In line with these findings, earlier research (39) showed that postgraduate dental students demonstrated higher levels of knowledge, attitudes, and practices related to sustainability compared to undergraduate students; however, these differences were not statistically significant.

### Perceptions of environmental impact in dental practice

Students predominantly identified patient care involving single-use materials as the principal contributor to dentistry's environmental burden. This perception accurately reflects current evidence: waste from single-use plastics, infection-control products, and disposable clinical items constitutes one of the largest waste streams in dental practice (16, 40). In light of the findings from our study, it is noteworthy that the primary contributor to dentistry's overall carbon footprint is CO₂ emissions associated with staff and patient travel (4), whereas the major source of general waste remains the extensive use of single-use plastics (41–44). Students' identification of dental equipment and materials as secondary sources of environmental impact is also consistent with reports indicating that equipment manufacturing, sterilization cycles, and procurement processes significantly contribute to dentistry's overall emissions (7).

### Approaches to reducing environmental impact: respondents' perspectives

The strategies most endorsed by students—reducing plastic disposables, adopting digital workflows, and implementing modern materials/technologies—mirror trends in sustainable dentistry research. Digital impressions, CAD/CAM workflows, and 3D-printing reduce transport emissions, material waste, and reliance on physical storage (45–47). Students' strong support for digitalization therefore reflects growing awareness of technological contributions to sustainability.

The gender difference observed—namely, male students expressing higher confidence in the sustainability benefits of modern dental materials—has, to our knowledge, not been widely reported in previous studies. It may reflect differences in exposure to material-science content or self-reported confidence levels. Similarly, Bachelor students' greater belief in the impact of digital technologies likely reflects greater curricular familiarity with emerging digital dentistry modules, which have become increasingly prominent in undergraduate programmes (24, 48–52).

### Perceived carbon footprint of dental procedures

Students' attribution of the highest carbon footprint to amalgam/composite fillings, radiographs, and acrylic dentures aligns with observations reported in the scientific literature and corresponds with published life-cycle assessments (LCA). In the same line, a relatively recent study (53) reported that dental amalgam exhibited the highest impact across most evaluated life cycle impact categories, whereas resin-based composites demonstrated the highest global warming potential and glass ionomer cements had the lowest impact across most categories; the authors suggested that adjustments to manufacturing processes and packaging materials could help reduce the environmental impacts associated with resin-based composites and glass ionomer cements, thereby supporting their use as viable alternatives to dental amalgam. Radiographic procedures, especially CBCT, contribute significantly through energy use and equipment manufacturing (54, 55). However, the considerable proportion of students, especially those in the Bachelor cohort—who selected “I don’t know (which dental procedures have the largest footprint)” highlights an important knowledge gap regarding the environmental impact of dental procedures. This finding is consistent with previous reports indicating limited formal education on environmental sustainability within dental curricula (25, 56). Notably, Master students more frequently associated porcelain crowns with a high environmental impact, possibly reflecting their greater clinical exposure, whereas Bachelor students more often selected precious metal crowns, suggesting they may be guided more by the material composition than by procedural complexity.

### Interest in sustainability education

The finding that 42% of students would choose an elective course on sustainable dentistry highlights substantial unmet educational demand. Recent studies report similar levels of interest, noting that while students value sustainability, they feel underprepared to translate principles into clinical practice (57, 58). There is a clear need for the structured and comprehensive integration of sustainability principles into dental education. Although awareness of these issues is increasing, notable gaps persist in faculty readiness, curricular consistency, and institutional commitment. Addressing these deficiencies will require coordinated action, including supportive policy frameworks, targeted faculty development initiatives, and the adoption of innovative teaching methods (59).

### Environmental attitudes and personal lifestyle behaviors

The majority of students (69%) expressed concerns about environmental issues and had made personal lifestyle changes. This reflects a general trend among young adults, who often express concern about the environment but struggle with a gap between knowledge and action (57). Gender differences were observed, with women more likely to express concern about environmental impact, consistent with sustainability studies across various educational fields (35). Male and Master students reported being more involved in activism for social causes, suggesting differing motivations or priorities within the concept of sustainability. Behavioral data showed that students most frequently took simple, low-cost environmental actions (turning off lights, walking/cycling, recycling). These findings parallel previous studies work showing that individuals tend to choose accessible pro-environmental behaviors even when more impactful actions (e.g., reducing consumption, using energy-efficient technologies) are less consistently adopted (60). Interestingly, female students recycled waste significantly more often than males, reflecting persistent gender trends in environmental behavior (35, 61, 62). These differences suggest that sustainability education in dental schools should potentially not only address professional behavior, but also the development of a broader environmental identity and sense of responsibility.

### Implications for dental education and policy

Taken together, the results demonstrate that the European dental students who participated in this study are aware of sustainability and motivated to engage with it, yet they lack standardized education and clear understanding of the environmental consequences of specific dental procedures. Recent frameworks advocate the implementation of “green dentistry” modules, the integration of environmental science into materials science and clinical courses, and the adoption of interprofessional sustainability education (31, 63). Additionally, dental schools should lead by example in sustainable operations—such as digital record-keeping, waste reduction practices, and optimized sterilization protocols—to reinforce sustainability through experiential learning. Given that dental healthcare systems contribute substantially to global healthcare emissions, improving sustainability literacy among future clinicians is essential for the transition toward low-carbon healthcare (64, 65).

### Sustainability awareness in dentistry in the context of other healthcare professions

Placing the findings of the present study in a broader interprofessional context may further enhance their interpretability. Previous research in medical education has highlighted a lack of systematic sustainable healthcare teaching within medical curricula, despite clear student interest, uncertainty regarding responsibility for delivery, optimal pedagogical approaches, and potential gender-related differences in perceived importance, underscoring a persistent gap between educational intent and implementation (66). Similarly, studies among healthcare students have reported uneven awareness of healthcare's environmental impact and limited engagement with formal sustainability training, accompanied by substantial differences in knowledge levels, perceived responsibility, and educational exposure (67). Comparable patterns have also been observed among European radiographers, who, although recognizing the importance of environmentally responsible practice, demonstrate gaps in understanding sustainable operational initiatives within radiology departments (68). Collectively, these findings suggest that the variability in sustainability awareness observed among dental students may not be unique to dentistry, but rather reflect broader challenges in integrating sustainability across healthcare professional education.

### Study limitations

This study has several limitations that should be considered when interpreting the findings. The method of questionnaire distribution is a limitation of the study. Deans of dental schools in Europe were asked to distribute the questionnaire to their students through the ADEE newsletter, and the EDSA board was also asked to distribute the questionnaire to students at affiliated dental schools. Because of this distribution method, it is impossible to determine which dental schools distributed the questionnaire or how many students actually received the invitation, and a response rate cannot be calculated.

The uneven distribution in the number of respondents across the various participating dental schools has introduced a potential geographical imbalance in the responses. Some students did not complete the full questionnaire. It is also possible that students interested in sustainability are more likely to complete the questionnaire than those who find this topic less relevant, and also were more likely to complete the whole questionnaire. Furthermore, it's possible that some participating students may have given socially desirable answers or consulted sources while completing the questionnaire. This could lead to an overestimation of both the knowledge and engagement of dental students in sustainability. For the statistical analysis, Chi-square tests were used which assume that observations are independent and have large enough expected cell counts. Although the number of cell counts was sufficiently large in most cases, one can discuss whether the independence assumption may have been violated for items in the questionnaire with multiple responses (where 3 or more options can be selected). Some of the very small cell counts might have been better analyzed using Fisher's exact test.

Given the sampling approach and methodological constraints, this research could be considered exploratory in nature and its results may be interpreted as preliminary; therefore, the generalizability of the findings may be limited, highlighting the need for further longitudinal research with larger and more representative samples. Moreover, the study sample, although diverse in terms of European representation, relied on voluntary participation and may therefore reflect a degree of self-selection bias, potentially overrepresenting students already interested in sustainability. The uneven distribution between Bachelor and Master students, as well as the predominance of female respondents, may also limit the generalizability of the results to the broader population of dental students in Europe.

Additionally, the use of self-reported questionnaire data introduces the possibility of response bias, particularly in areas concerning attitudes and environmentally responsible behaviors.

### Future research

Future research should aim to include larger and more balanced cohorts across all academic stages, incorporate longitudinal designs to assess how sustainability awareness evolves during dental education, and integrate objective measures of knowledge and behavior. Qualitative approaches may also provide deeper insight into students’ motivations, barriers, and educational needs, supporting the development of evidence-based sustainability curricula in dentistry. Moreover, formal psychometric testing, including assessments of face and content validity and cross-cultural validation, was not conducted. Future research should incorporate formal pilot testing and validation procedures across diverse cultural and educational contexts to strengthen the robustness and generalizability of the instrument.

## Conclusions

1. European dental students participating in this survey demonstrated awareness of the importance of sustainability and recognized key environmental challenges associated with dental practice. Students identified high-impact activities such as the use of single-use materials and selected realistic strategies to reduce environmental burdens, including digitalization and waste reduction. However, significant knowledge gaps were still evident, particularly concerning the carbon footprint of dental procedures and the environmental impact of materials, highlighting the need for structured sustainability education within dental curricula.
2. Many students report behaving in ways which are environmentally friendly, though gender and academic stage influence specific attitudes and actions. The substantial interest in elective sustainability courses underscores an educational opportunity for dental schools to integrate environmental responsibility into mainstream training.
3. Overall, these findings could contribute to ongoing discussions regarding policy and curriculum development, potentially supporting efforts to equip future dental practitioners with the knowledge, skills, and mindset needed to engage with sustainable healthcare practices.

## Data Availability

The original contributions presented in the study are included in the article/Supplementary Material, further inquiries can be directed to the corresponding author.
