# Supplementary material for: Are tomorrow's dentists ready for a sustainable future? Insights from a European student survey
Source: Front Dent Med. 2026 Jun 8;7:1788789. doi: 10.3389/fdmed.2026.1788789 (PMC13284150; doi:10.3389/fdmed.2026.1788789)
Supplement: Supplementary file 1 [file Datasheet1.pdf]

Dear student,

You are invited to participate in a study conducted by the faculty members of the Faculty of Dentistry at the Carol Davila University of Medicine and Pharmacy in Bucharest, Romania, and the Academic Centre for Dentistry Amsterdam (ACTA), the combined Dental Faculty of the Vrije Universiteit Amsterdam and the University of Amsterdam, Netherlands. The study is approved by the Scientific Research Ethics Committee of „Carol Davila" University of Medicine and Pharmacy, Bucharest, Romania.

The study aims to assess the level of knowledge about sustainability in dental medicine among dental students across Europe. The study will be conducted online. As a participant, you will complete a Qualtrics questionnaire with 16 items, divided into three sections, including multiple-choice and open-ended questions.

Participation in the study is voluntary and anonymous. The data will be collected at ACTA (Vrije Universiteit of Amsterdam, Netherlands), and appropriate data protection measures have been be implemented. You will not receive financial compensation for participating in the study. Choosing not to participate in this study will not affect you in any way.
By completing the questionnaire, you are consenting to participate in the study.

1. Which dental faculty/dental school are you from?
    - Name of dental school/ dental faculty
    - City
    - Country

2. What year are you in?
    - Year 1
    - Year 2
    - Year 3
    - Year 4
    - Year 5
    - Year 6

3.What best describes your gender?
    - Male
    - Female
    - Other
    - Prefer not to say

4. What age are you(in years)?

**Your perspective on sustainability issues in dentistry**

5. Which definition of sustainability below is, in your opinion, the most relevant to the dental profession?

- Meeting the needs of dental patients and the present population without compromising the ability of future generations to meet their own needs

- Taking care of the three "pillars": the environment, society, and the economy, which are interdependent and interconnected
- Minimizing the health-related impact of climate change and environmental degradation
- All of the above

6. How important is it for dentists to be aware of sustainability practices in their sector?
- 1 – sustainability is not an issue for dentists
- 2
- 3
- 4
- 5
- 6
- 7
- 8
- 9
- 10 – sustainability is an absolute priority for dentists

7. Which activity associated with dentistry has the highest adverse environmental impact, in your opinion?

- Use of specialist materials
- Dental equipment
- Patient care aspects such as single-use cups and materials
- Patient paperwork & records
- Dental office lighting & energy
- Commute and use of transport to and from the dental office by the patients
- Other (please specify)

8. Select the top 3 activities you consider to be the most effective in improving environmental impact:

- Use of modern dental materials
- Use of modern techniques and technologies (3D Printing, CAD/CAM, etc.)
- Digital data management & records
- Reducing the use of plastic medical disposals
- Reducing water and electricity consumption

- Educating staff and patients
- Other (please specify)

9. A carbon footprint is the total amount of greenhouse gas emissions caused by an individual, event, organization, service, place, or product.
What are, in your opinion, the top 3 dental procedures with the largest carbon footprint (select 3):

- Scale and polish
- Amalgam and composite fillings
- Acrylic dentures
- Radiographs
- Extractions
- Non-precious metal crowns
- Fluoride varnish
- Endodontic treatment
- Precious metal crowns
- Metal dentures
- Fissure sealants
- Porcelain crowns
- I don’t know

10. What are the opportunities for the dental profession to improve its environmental impact?

11. Are there any initiatives with regard to sustainability that you would like to be undertaken by your dental school?

- Yes (please specify)
- No

12. If there was an optional course (elective) on sustainability in dentistry, would you be interested in attending?

- Yes
- No
- I am undecided, I need more information

13. How would you describe your lifestyle (select all that apply):

- I am very aware of my environmental impact, and it is a big part of all my decisions
- I am very active in supporting social causes that are important to me
- I am worried about the environment, and I have made some changes in my lifestyle, however, I probably could do more if I knew what else to do
- I am not doing anything about my environmental impact, but I would like some help to get started
- I am not concerned about my environmental impact

14. Are you currently involved in any sustainability initiatives or organizations (on campus or outside)?

- Yes (Please specify)
- No

15. Please select the choices you try to make regularly (select all that apply):

- Walk or cycle to work/university campus
- Use only public transport
- Turn off the lights when I’m not using them
- Use energy-efficient light bulbs
- Buy organic food
- Recycle waste
- Buy less
- Buy used/refurbished goods
- Other

16. Are there any other comments you would like to share?
